# Plant essential oils and potassium metabisulfite as repellents for *Drosophila suzukii* (Diptera: Drosophilidae)

**DOI:** 10.1038/srep21432

**Published:** 2016-02-19

**Authors:** Justin M. Renkema, Derek Wright, Rose Buitenhuis, Rebecca H. Hallett

**Affiliations:** 1 School of Environmental Sciences, University of Guelph, 50 Stone Rd. E., Guelph, Ontario, Canada, N1 G 2W1; 2 Vineland Research and Innovation Centre, 4890 Victoria Ave. N., Box 4000, Vineland Station, Ontario, Canada, L0R 2E0

## Abstract

Spotted wing drosophila, *Drosophila suzukii*, is a globally invasive pest of soft-skinned fruit. Females oviposit into ripening fruit and larvae cause direct destruction of tissues. As many plant essential oils are permitted food additives, they may provide a safe means of protecting fruit from *D. suzukii* infestation in both conventional and organic production systems. Twelve oils and potassium metabisulfite (KMS) were screened in the laboratory as repellents for *D. suzukii* flies. Most essential oils deterred *D. suzukii* flies from cotton wicks containing attractive raspberry juice. Peppermint oil was particularly effective, preventing almost all flies from contacting treated wicks and remaining 100% repellent for 6 d post-application. Thyme oil was unique because it caused high male mortality and reduced the number of responding flies compared to other oils. KMS was not found to be repellent to *D. suzukii*, but may have fumigant properties, particularly at high concentrations. Peppermint oil appears to be the best candidate for field testing to determine the effectiveness and feasibility of using essential oils as part of a push-pull management strategy against *D. suzukii*. This is the first time that essential oils have been evaluated and proven effective in preventing fruit-infesting flies from contacting attractive stimuli.

*Drosophila suzukii* Matsumura (Diptera: Drosophilidae) is a globally invasive, multivoltine pest of numerous soft-skinned temperate fruit crops^1^. Unlike most other drosophilids, female flies have a serrated ovipositor that allows them to lay eggs in ripe and ripening fruit^2^,^3^. Developing maggots cause softening of fruit and may promote accelerated decomposition, rendering fruit unmarketable^3^. Under heavy infestations, up to eighty percent yield loss has been reported^4^, and 20 and 37% losses in revenue were estimated in untreated California strawberries and raspberries, respectively^5^. *D. suzukii* is indigenous to Japan and is recorded from other Asian countries^6^. The invasion of other regions by *D. suzukii* was first noted in California and Spain in 2008; it has since spread across North America and Europe and has most recently been found in South America^6^,^7^,^8^,^9^.

Insecticides are available for effective control of *D. suzukii*^10^,^11^,^12^ but due to both the relatively short generation time of *D. suzukii* and limited residual control afforded by insecticides (5–14 days), frequent applications may be necessary to maintain low pest levels^11^. Field sanitation is recommended, including removal of overripe fruit from fields and from fruiting alternate host plants in areas adjacent to fields^3^,^7^,^13^. Exclusion netting has been found to be effective for protecting fruit from *D. suzukii* infestation^14^. Natural enemies including endemic parasitoids, commercially-available predators, and entomopathogenic nematodes and fungi have been tested against *D. suzukii*^15^,^16^,^17^,^18^,^19^, but to reduce reliance on insecticides and improve *D. suzukii* control, these and new tools for management need to be developed.

It is not yet clear how *D. suzukii* locate suitable hosts, although volatile leaf odors are likely involved^20^, but compounds that deter flies from fruit or reduce contact time with fruit may be useful in a management program^21^,^22^. Aromatic plants produce characteristic blends of volatile organic compounds that can be concentrated as essential oils from leaves or other plant parts by steam distillation^23^. Many essential oils have recently come into focus as repellents, antifeedants, oviposition deterrents, or toxicants for managing plant, human or animal nuisance pests^24^,^25^. Natural products, including essential oils, are perceived as posing a lower risk to the environment and humans compared to synthetic compounds^26^, although safety is dependent on biological properties of and exposure to chemicals that are not always consistent with their origin^27^. Second, the large diversity and redundancy of phytochemicals in a single essential oil, including many mono- and sesquiterpenes, can improve control efficacy and reduce selection pressure and resistance development in pests^25^,^28^,^29^. Finally, there is an increased availability of essential oils, as many are registered as flavouring or perfuming agents^24^ Essential oils may have potential for use in organic small fruit production systems, as there are currently limited management options for *D. suzukii* available.

As crop protection agents, essential oils have been widely tested as repellents or fumigants for stored product pests^30^ and as repellents for biting or nuisance pests, mainly flies, of livestock^31^. In the latter, essential oils act to prevent insects from flying to, landing on, or biting skin. House flies, *Musca domestica* L., are repelled by basil, lemongrass, lavender, peppermint, ginger, geranium, and elemol (osage orange) oils^32^,^33^,^34^, and eucalyptus oil was used as a fumigant against the horn fly (*Haematobia irritans*, L.)^35^. Essentials oils can also be effective repellents of herbivorous pests, including lavender and pennyroyal (mint) for codling moth larvae (*Cydia pomonella* (L.))^36^, lavender, juniper and citronella against red bud borer midges (*Resseliella oculiperda* (Rübsaamen)) on apple saplings^37^, and common thyme and winter savory for Western flower thrips (*Frankliniella occidentalis* (Pergande))^38^. Peppermint, thyme, lavender and rosemary oils caused mortality in *Drosophila auraria* flies^39^, but to our knowledge, essential oils have not been tested as repellents or deterrents to prevent fruit infesting flies from contacting host fruits or surfaces containing attractive stimuli.

In addition to essential oils, potassium metabisulfite (KMS) may be useful as a repellent for *D. suzukii*. KMS (K_2_S_2_O_5_) produces sulfur dioxide when dissolved in water^40^ and is a common additive in food and the wine-making process. Sulfur dioxide acts as an antimicrobial and antioxidant, preventing browning by inhibiting bacterial growth during wine-making^41^. In the only report on repellency towards insects, KMS repelled *Harmonia axyridis* (Pallas) in a Y-tube olfactometer and reduced numbers of beetles on grape vines^42^. KMS is not registered for field-use worldwide, but it may be useful for controlling *Botyritis* in vineyards^40^.

The objective of this study was to screen 12 essential oils and KMS as repellents for *D. suzukii* flies in the laboratory. A bioassay allowed male and female flies to choose between an attractant or the attractant plus an essential oil. Essential oils tested were selected from those which may have applicability in the field (eg., low-cost, high persistence, or from plants that could be grown in or near fruit fields in temperate regions). Due to its high volatility, KMS was assessed in a no-choice trapping assay. Our results are a first step towards selecting and further testing of repellents for developing a push-pull management strategy against *D. suzukii*.

## Results

**Figure 1:**
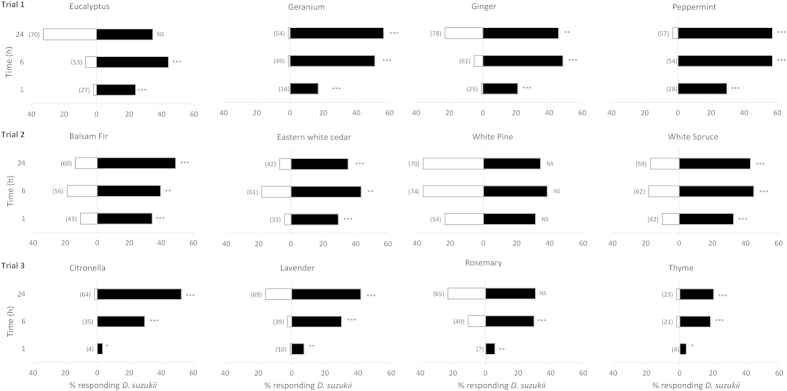
Percent male *Drosophila suzukii* choosing cotton wicks with raspberry juice (filled bars) or raspberry juice + essential oil (empty bars) at 1, 6 and 24 h after start of experiment (Exp. 1). Numbers of flies responding given in parentheses. Significant differences (G test): ***(*P *< 0.001), **(*P *< 0.01), *(*P *< 0.05), ‘NS’ no significant difference (*P *> 0.05).

In Exp. 1, all essential oils, except white pine, showed a significant level of repellency to male *D. suzukii* (Fig. 1). Few male flies chose geranium, peppermint, citronella, or thyme oil-treated wicks throughout the duration of the experiment, whereas rosemary and eucalyptus oils did not repel male flies after 24 h. Eastern white cedar, balsam fir, white spruce, ginger, and lavender oils were significantly repellent after 24 h, but approximately 20% of flies settled on wicks treated with these oils.

**Figure 2:**
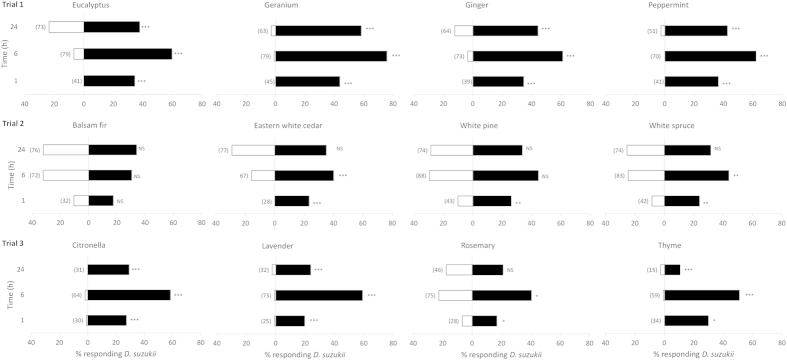
Percent female *Drosophila suzukii* choosing cotton wicks with raspberry juice (filled bars) or raspberry juice + essential oil (empty bars) at 1, 6 and 24 h after start of experiment (Exp. 1). Numbers of flies responding given in parentheses. Significant differences (G test): ***(*P *< 0.001), **(*P *< 0.01), *(*P *< 0.05), ‘NS’ no significant difference (*P *> 0.05).

All essential oils, except balsam fir, showed a significant level of repellency to female *D. suzukii* (Fig. 2). Few female flies chose geranium, peppermint, citronella, lavender, or thyme oil-treated wicks throughout the duration of the experiment, whereas eastern white cedar, white pine, white spruce, and rosemary oils did not repel flies after 6 or 24 h. Ginger and eucalyptus oils were significantly repellent after 24 h, but 10–20% of females settled on wicks treated with these oils.

There were few differences among essential oils in the percent of flies responding or dying during this experiment (Table 1). There were fewer responding female flies in the peppermint oil than geranium oil treatments in group one, and thyme oil caused higher male fly mortality than the other three oils in group three (Table 1).

**Figure 3:**
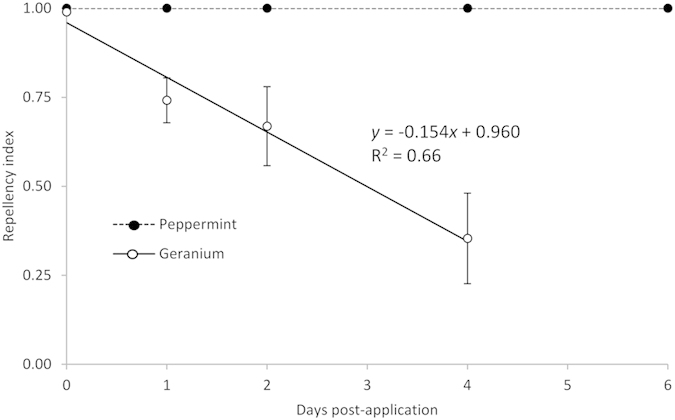
Response of *Drosophilia suzukii* female flies to peppermint (*Mentha* x *piperita*) and geranium (*Pelargonum asperum*). Essential oils were applied at 30 mg/wick up to 4 or 6 days before the start of Exp. 2, and flies were given a choice of a cotton wick with raspberry juice or a wick with juice and an essential oil. Repellency index is the proportion of flies on wicks without essential oils out of all flies that chose either wick.

In Exp. 2, peppermint oil remained 100% repellent to female flies for up to 6 d after application on the wick (Fig. 3). Repellency of geranium oil to female flies decreased as time since application increased (*F*_1,14_ = 27.9, *P *< 0.001), decreasing to about 35% by 4 d post application (Fig. 3).

**Figure 4:**
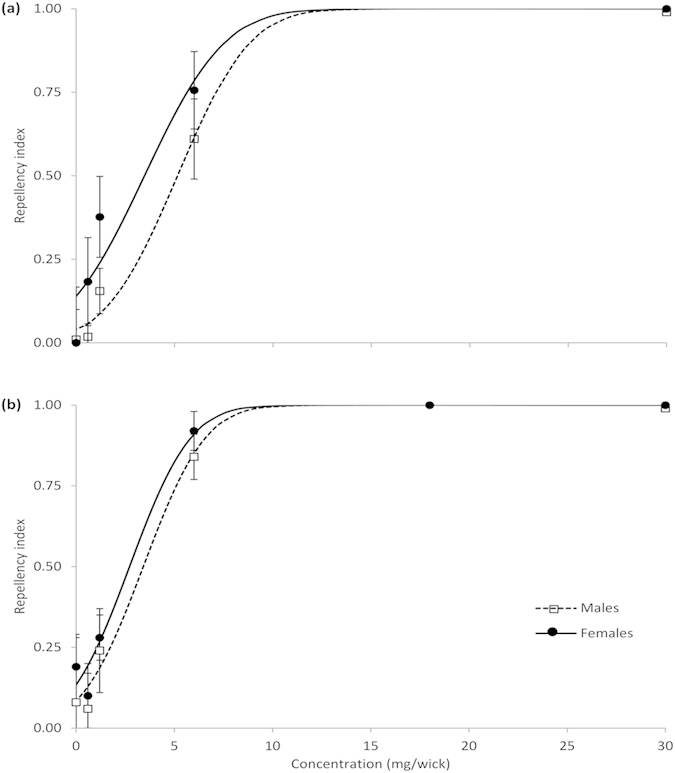
Response of *Drosophilia suzukii* male and female flies (means ± SE and probit analyses lines-of-best-fit) to increasing concentrations of (**a**) peppermint (*Mentha* x *piperita*) and (**b**) geranium (*Pelargonum asperum*). Flies were given a choice of a cotton wick with raspberry juice or a wick with juice and an essential oil, Exp. 3. Repellency index is the proportion of flies on wicks without essential oils out of all flies that chose either wick.

In Exp. 3, repellency of peppermint and geranium oils to male and female flies was dependent on concentration (peppermint, males: χ^2^ = 48.9, *P *< 0.001, females: χ^2^ = 26.6, *P *< 0.001; geranium, males: χ^2^ = 29.9, *P *< 0.001, females: χ^2^ = 65.6, *P *< 0.001) (Fig. 4). Concentrations required to repel 95% of flies were 1.1 times greater for males than females for both peppermint and geranium oils, and 1.3 times more peppermint than geranium oil was required to repel both male and female flies (Table 2).

### Essential oil release rates

Volatility, measured as decrease in weight over 96 h, varied by essential oil (*F*_11,33_ = 66.9, *P *< 0.001). Volatility was highest in tree-derived and rosemary essential oils, although volatility of Eastern white cedar oil was lower than for other tree oils (Table 3). Peppermint oil was least volatile, followed closely by geranium, citronella, and lavender oils. Release rates of all oils tended to decline over time, although differences in release rates between the first 6 h and the last 72 h were much greater for some oils (e.g., thyme, lavender) than others (e.g., ginger, peppermint).

### KMS no-choice bioassay

**Figure 5:**
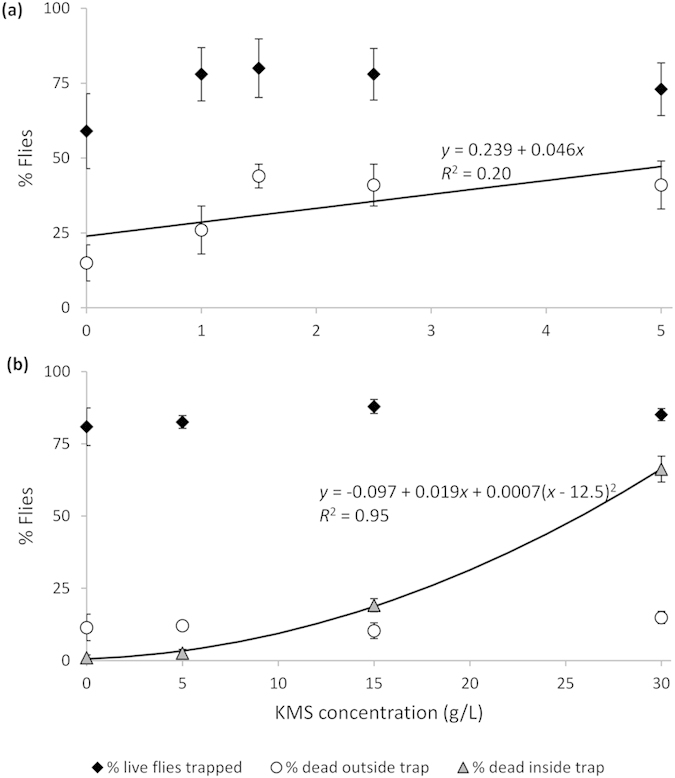
Response of *Drosophila suzukii* (±SE) to traps containing two red raspberries and cotton wicks containing 1.5 mL of potassium metabisulfite (KMS) solution in the laboratory in (**a**) Exp. 4 and (**b**) Exp. 5.

In Exp. 4, KMS concentration did not affect the percent of live flies trapped (*F*_1,23_ = 0.35, *P *= 0.558), but increasing KMS concentration caused an increased percent of dead flies outside traps (*F*_1,23_ = 5.79, *P *= 0.025) (Fig. 5), even though none of the trapped flies were dead. In Exp. 5, KMS concentration affected neither the percent of live flies trapped (*F*_1,22_ = 0.84, *P *= 0.3683) nor the percent of flies outside traps that were dead (*F*_1,22_ = 0.64, *P *= 0.432), but increasing KMS concentration caused an increase in the number of trapped flies that died (*F*_1,22_ = 198.41, *P *< 0.001).

## Discussion

In these experiments most essential oils deterred *D. suzukii* male and female flies from wicks containing attractive, fresh raspberry juice. Peppermint oil was particularly effective, preventing all but a few flies from contacting treated wicks and remaining 100% repellent for up to 6 d after it was applied to wicks. Peppermint oil consists mainly of two monoterpenoids: menthol and menthone^43^. Mint oils and their constituents were the best of 86 essential oil repellents tested against German cockroaches (*Blattella germanica*)^44^, and peppermint oils have shown strong and prolonged repellent action against flies, including mosquitoes^45^, house flies^30^,^31^ and other nuisance flies^46^. Prolonged repellency of peppermint oil may be due to its consistently low release rate. It was the least volatile of all tested essential oils, losing only 1.2% of its weight over 96 h. However, in at least one study, after aging for 72 h peppermint oil at multiple concentrations was not more repellent than other essential oils to the housefly, *Musca domestica* L.^31^.

Mint oils have also been reported as good adulticides and larvicides against insect pests^30^, and pennyroyal oil, *Mentha pulegium* L., caused high *D. auraria* mortality^39^. We did not find greater mortality of *D. suzukii* adults due to peppermint oil. There were fewer responding female flies in assays with peppermint oil than with geranium oil, suggesting that peppermint oil may negatively affect flies at a greater distance from its release point than geranium oil. Peppermint oil appears to be a good candidate for testing under field conditions, where persistence of repellency and distance of repellency from ripe fruit will be important factors determining the effectiveness and feasibility of using essential oils as part of a management strategy for *D. suzukii*.

Amounts of peppermint and geranium oils estimated to achieve 95% repellency of female and male flies were similar, ranging from 6.8–9.9 mg of oil per wick or 0.39–0.57 mg cm^2 −1^ based on the wick surface area. Studies on third-instar larvae of small dipterans have found an LC_50_ of 0.70 mg cm^2 −1^ of peppermint oil for *Camptomyia cortacalis* (Diptera: Cecidomyiidae)^47^ and an LD_50_ of 2.1 and 1.1 μL of pennyroyal and spearmint oils, respectively, for *Drosophila melanogaster*^48^. Konstantopoulou^43^ found 100% mortality of *D. auraria* when eggs and adults were exposed to *M. pulegium* but stated only that 1–20 μL of an essential oil was used. The estimated amount of peppermint oil on the exposed part of the wick required to achieve 95% repellency in our study was 4.5–6.7 μL. It is difficult to estimate the actual dose experienced by flies as we do not know whether the wicking action and evaporation rate of raspberry juice changed and affected the oil concentration during the experiment. Distances at which oils are repellent and concentrations required to achieve repellency and deter oviposition in the field will need to be determined.

Thyme oil was unique among oils tested because it caused higher mortality of male flies than other oils, and it reduced the number of responding males and females to 56–65% and 75–81%, respectively, of that of flies responding in arenas with citronella, lavender, and rosemary (Trial 3). Thyme essential oil constituents, particularly the monoterpene thymol from the chemotype of *Thymus vulgaris* used in this experiment, have strong toxic effects on other insect pests, including southern green stink bug, *Nezara viridula* (L.), nymphs and adults^49^, larval tobacco cutworm, *Spodoptera litura* Fab.^50^, and third-instar gall midge *C. cortacalis* (Diptera: Cecidomyiidae)^47^. Toxicity of thymol has been shown in *D. melanogaster* to be related to interference in tyramine receptor cascades that are involved with cAMP and calcium at the molecular level^51^. It also binds to GABA receptors associated with chloride channels, disrupting the function of GABA synapses^52^. Excellent levels of repellency or deterrence using thyme have also been recorded against pest insects, including Western flower thrips adults from leaf discs^36^ and *Culex pipiens pallens* Coquillet mosquitoes^53^. As with peppermint oil, thyme oil appears to be a good candidate for further testing, and thyme oil may soon be available as an insecticide in the European Union^24^.

Our results show that KMS is not repellent to *D. suzukii* flies but may have fumigant properties, particularly at high concentrations. At lower concentrations (Exp. 4), there was a small but significant increase in fly mortality from less than 25% in controls to about 40% at 5 g L^−1^. At higher concentrations (Exp. 5), 10–15% of flies died outside traps at all concentrations, but mortality in traps reached 60% at 30 g L^−1^, a significant increase from no mortality in traps at 0 and 5 g L^−1^. KMS is highly volatile in solution, releasing half its weight in SO_2_ and repelling *H. axyridis* within 1 min after the start of assays^42^,^54^. Sulfur dioxide has been shown to be acutely toxic to omnivorous leafroller (*Platynota stultana* Walshingham), providing protection to packed table grapes at low doses in combination with low temperature over long periods^55^. Although no concentration of KMS repelled flies from traps after 24 h, likely due to rapidly declining concentration, sufficient SO_2_ was produced to kill flies, particularly within traps where SO_2_ would have been more concentrated. It is also possible that once inside traps, flies died by contact with or ingestion of KMS on wicks. KMS may be more effective as a fumigant in enclosed spaces than as a repellent in open fields and could be investigated against *D. suzukii* larvae as a post-harvest alternative to methyl bromide^56^.

This is the first time that essential oils have been shown to be effective in preventing fruit-infesting flies from contacting attractive host stimuli. Essential oils from the same plant species may vary in their constituent chemical composition due to production practices or plant material sources and thus affect their bioactivity. Using essential oils or their active components (see gas chromatograph results at www.aliksir.com for most of the essential oils used in these experiments) as deterrents for *D. suzukii* and alternatives to synthetic insecticides is desirable because they pose little or no risk to mammalian health or beneficial insects^23^. As many essential oils are permitted food additives, repellent essential oils may provide a safe means of protecting fruit from *D. suzukii* infestation applicable in both conventional and organic production systems.

Other compounds tested as *D. suzukii* deterrents include geosmin, 1-octen-3-ol, and butyl anthranilate in the laboratory; 1-octen-3-ol reduced infestation in fruit in the field by 40–50%^57^,^58^. Further research will be needed to compare these molecules with those in essential oils and to test blends of active compounds. Factors such as compound dose, persistence, volatility, availability and cost will be important, but determining effective dispersal mechanisms for field application of repellents will be crucial to success. Products that are phytotoxic or compromise fruit quality cannot be applied directly to ripening fruit, and small dispensers required near each bunch of fruit^58^ may not be feasible on a large scale. Methods for dispensing insect pheromones (e.g., biopolymer flakes, rubber septa, aerosols) may be appropriate and adaptable for repellent dispersal. If repellents are to be used in the field, then they will likely need to be part of a push-pull management **s**trategy to provide sufficient control. Mass trapping, attract-and-kill, or other ‘pull’ strategies also need to be further developed and integrated with other cost-effective management practices in order to achieve the high-level of control needed for this important pest^59^.

## Methods

### Fly colony

*Drosophila suzukii* flies used in bioassays were from a laboratory colony established in September 2012 at the University of Guelph from infested raspberries and blackberries collected at a commercial farm near Halton Hills, ON, Canada (43 N 34′ 43; 79 W 57′ 38″). Flies were kept in Plexiglas^®^ cages (26 × 26 × 26 cm), with mesh backings and sleeves for access, at 22–23 ^o^C, 20–30% RH and 16:8 h L:D. Flies were provided with moist cotton batting and fed and reared on diet prepared by combining: water (4 L), agar (45 g), cornmeal (125 g), white sugar (200 g) and nutritional yeast (70 g) with propionic acid (17.7 mL) and methyl paraben (3.3 g) dissolved in 95% ethanol (33.3 mL). The mixture was boiled and then cooled before pouring into Petri dishes (9 mm diameter). Flies that were 5–8 d old were used for experiments. Flies were separated by sex and held without food for 20 h and without water for the final 2 h before the start of the experiments.

### Essential oil choice bioassays

Essential oils tested in repellency bioassays were geranium (*Pelargonium asperum* or *P. graveolens* (L.) L’Her ex Ait., cv. Bourbon or Rosat), peppermint (*Mentha* x *piperita* L.), ginger (*Zingiber officinale* Roscoe), eucalyptus (*Eucalyptus radiata* Spreng.), citronella (*Cymbopogon winterianus* Jowitt), lavender (*Lavandula angustifolia* Mill.), rosemary (*Rosmarinus officinalis* L.), thyme (*Thymus vulgaris* L. (thymol chemotype)), eastern white cedar (*Thuja occidentalis* L.), balsam fir (*Abies balsamea* (L.) Mill.), white spruce (*Picea glauca* (Moench) Voss), and white pine (*Pinus strobus* L.); all obtained from Aliksir Inc. (Grondines, QC, Canada). Essential oil density was determined by weighing three samples of 10 μL (Table 3). Stock solutions of 15 g L^−1^ were prepared with acetone as the solvent. Varying concentrations (0.3–30 g L^−1^) were prepared for experiments with geranium and peppermint oils.

Choice bioassays were conducted in arenas consisting of clear, plastic containers (33.3 × 20.3 × 12.1 cm) with tight fitting lids (KIS Omni Shoe Box, a.b.m. Canada Inc., Milton, Ontario). Fine white mesh covered ventilation holes (2.2 cm diam.) made singly on each vertical side (7.0 cm from the bottom) and three holes in the lid. In each arena, two glass specimen vials (12 mL) were placed 18.7 cm apart and 7.3 cm from the ends of the container. Vials were secured to the bottom of containers with small pieces of mounting putty (Lepage, Henkel Canada Corp., Mississauga, ON, Canada). A cotton dental wick (3.5 cm long; No. 2 medium, Mydent International, Hauppauge, New York) and 10 mL of raspberry juice was added to each vial. A second dental wick was treated with either an essential oil (2 mL acetone solution containing 30 mg of essential oil) or acetone only (2 mL) and placed in a fumehood for one hour to allow acetone to volatilize. Raspberry juice (2 mL) was applied to each wick before it was put in a vial; the wick rested on the wick already in the vial and protruded 2 cm above the rim of the vial. Raspberry juice was prepared by heating fresh, store-purchased red raspberries (400 g) mixed with distilled water (250 mL) and pectinase (1.5 g; Vineco International Products, St. Catharines, Ontario) at 45 ^o^C for 1 h. The mixture was strained under vacuum through a paper coffee filter, and juice was stored overnight in a refrigerator. After each experiment, arenas and vials were washed with water and soap (Sparkleen, Fisher Scientific Co., Pittsburgh, Pennsylvania), air-dried and wiped or rinsed with hexane.

Arenas were arranged in a completely randomized design on a table covered with white paper. Three 60 W incandescent bulbs were hung 1 m above the table at 16:8 h L:D with photophase beginning at 0600 h. Trials began at 1100 h by placing plastic vials (70 mL) containing approximately 20 flies upright in the center of the container and removing lids so that flies could walk or fly from vials. The number of responding flies (those on treated or control wicks) and the number alive or dead in the arena and release vial were counted at 1, 6, and 24 h after flies were introduced.

#### Experiment 1

Essential oils were tested in three trial periods. The first trial tested peppermint, geranium, ginger, eucalyptus, the second trial tested eastern white cedar, balsam fir, white spruce, white pine, and the third trial tested citronella, lavender, rosemary, thyme with female and male flies separately. Each oil was replicated six times for each sex, except peppermint, geranium and group 2 oils that were replicated five times for males due to lower than expected numbers of available flies.

#### Experiment 2

Persistence of repellency to female flies was assessed by treating wicks 0, 1, 2, 4 d or 0, 1, 2, 4, and 6 d before the start of the experiment with geranium and peppermint oil, respectively. Wicks were held on aluminum foil in a fumehood at 22 °C ± 1 °C for aging. There were four geranium and five peppermint oil replicates.

#### Experiment 3

The effect of geranium and peppermint oil concentrations on repellency to male and female flies was evaluated by treating wicks with 0, 0.6, 1.2, 6, and 30 mg of peppermint and geranium oil; 18 mg of geranium oil was also included in assays with females. Each peppermint and geranium concentration was replicated five times, except geranium concentrations were replicated four times for females because of the extra concentration and a shortage of female flies.

### Essential oil release rates

Release rates of essential oils were determined by measuring weight loss from 2 mL polypropylene vials (Fisher Scientific, Toronto, Ontario), each with a 3 mm hole in the lid. Vials were loaded with 400 μL of an undiluted essential oil and placed in a fume hood at 22 °C ± 1 °C. Vials (three per oil) were weighed 0, 2, 6, 12, 24, 48, 72, and 96 h after loading.

### KMS no-choice bioassay

Solutions of 1.0, 1.5, 2.5, 5.0, 15.0, and 30.0 g KMS (Vineco International Products, St. Catharines, Ontario) per litre of distilled water were prepared 1 h prior to the start of the experiments.

A no-choice trapping bioassay was conducted in arenas consisting of upright clear Plexiglas^®^ cylinders (31 × 8.8 cm) with ends covered by fine white mesh held in place at the top of the cylinder by an elastic band and glued to the base of the cylinder. There were two ventilation holes (5 cm) covered with fine white mesh 20.3 cm from the bottom of each cylinder. Traps were plastic jars (7.3 × 4.8 cm) with aluminum foil secured by an elastic band over the jar opening. A cut microcentrifuge tube (1.5 mL) was inserted through the middle of the foil. Two store-purchased red raspberries (6.9 ± 1.4 g) and a cotton wick that was treated with KMS solution (1.5 mL) or untreated (1.5 mL water) were placed in each trap. One trap was placed in each cylinder. Cylinders were arranged in a completely randomized design on a table under the same conditions as described above for choice bioassays.

#### Experiments 4 and 5

KMS was tested at four concentrations between 1–5 g L^−1^ with five replicates (Exp. 4). Because there was no repellent effect of KMS at these concentrations, Exp. 5 was conducted using concentrations of 5, 15, and 30 g L^−1^ with six replicates. For each experiment, 25–35 flies (approximately 1:1 sex ratio) were released into each cylinder. After 24 h, the number of trapped flies and the number of dead flies inside and outside traps were counted.

### Data analysis

For Exp. 1, numbers of *D. suzukii* on treated and untreated wicks were compared using goodness-of-fit *G*-tests. The pooled *G*-test statistic is presented as results of each essential oil for either male or female flies were consistent between replicates (heterogeneity *G*-test, *P* > 0.05)^60^. Analysis of variance (ANOVA) was used to test effects of essential oils by groups on the percent responding flies at 6 h (percent on treated wicks + percent on untreated wicks) and percent fly mortality at 24 h. Percentages were square-root transformed to normalize residuals. ANOVA was also used to evaluate differences between release rates of essential oils over 96 h. All means were separated using Tukey’s HSD test.

For Exps. 2 and 3, a repellency index (RI)^31^ was calculated at 6 h after the start of the experiment:

1. RI = (*flies on treated wicks − flies on untreated wicks)*/*(flies on both wicks)*

Linear regression was used to evaluate geranium oil persistence, where *Y* was the repellency index and *X* was the age of the treated wicks in days. For male and female flies, probit analysis was used to generate concentration–repellency regressions for peppermint and geranium oils. Concentrations that achieved 95% repellency (EC 95), confidence limits, slopes, and goodness-of-fit χ^2^ vales were determined.

For Exps. 4 and 5, linear regression was used to assess repellency, where *Y* was the percent trapped flies or percent dead flies inside and outside traps and *X* was the KMS concentration (g L^−1^). A quadratic regression was also fit to the percent dead flies inside traps in experiment five. Significance of slopes (deviation from zero) and coefficients of determination (*R*^2^) are reported for regressions.

JMP software^61^ was used for ANOVAs, regressions, and PROBIT analyses. In all cases, α = 0.05.

## Additional Information

**How to cite this article**: Renkema, J. M. *et al.* Plant essential oils and potassium metabisulfite as repellents for *Drosophila suzukii* (Diptera: Drosophilidae). *Sci. Rep.* **6**, 21432; doi: 10.1038/srep21432 (2016).

## Figures and Tables

**Table 1:** Percent (±95% CI) *Drosophila suzukii* responding flies (on cotton wicks with raspberry juice or with raspberry juice + essential oil) and mortality during Exp. 1.

Trial	Essential oil	% responding flies (6 h)		% dead flies (24 h)	
		Male^a^	Female^b^	Male^c^	Female^d^
1	Eucalyptus	59.3 (45.0−75.5)	69.4 (61.7−77.6) **ab**	12.9 (7.0−20.7)	2.4 (0.1−8.3)
	Geranium	57.1 (41.9−74.7)	78.3 (70.1-86.9) **a**	12.2 (5.9− 20.6)	0.7 (0.3-4.6)
	Ginger	61.4 (46.9−78.0)	69.0 (61.3−77.2) **ab**	9.6 (4.6−16.4)	6.0 (1.3−14.4)
	Peppermint	64.1 (47.9−82.7)	62.0 (54.7−69.7) **b**	12.8 (6.4−21.4)	1.1 (0.1−5.7)
2	Balsam fir	67.1 (46.9−82.6)	70.9 (58.7−84.2)	8.2 (4.1−13.6)	4.1 (1.7−7.5)
	Eastern white cedar	79.1 (62.8−92.6)	58.5 (47.5−70.7)	14.7 (9.0−21.8)	2.1 (0.5−4.7)
	White pine	91.7 (78.2−100.0)	78.9 (66.1−93.0)	14.0 (8.5−20.9)	4.1 (1.7−7.5)
	White spruce	82.9 (67.6−95.8)	77.3 (64.4−91.1)	11.2 (6.4−17.5)	5.9 (3.0−10.0)
3	Citronella	30.2 (17.2−46.9)	68.4 (52.9−85.7)	0.2 (0.1−3.6) **b**	4.6 (0.3−14.1)
	Lavender	32.4 (18.8−49.6)	63.3 (48.5−80.1)	2.1 (0.1−8.6) **b**	1.8 (0.1−8.8)
	Rosemary	38.3 (23.3−56.9)	68.6 (53.2−86.0)	1.0 (0.2−6.3) **b**	7.3 (1.2−18.6)
	Thyme	13.6 (5.4−25.4)	51.2 (38.0−66.4)	22.0 (10.2−38.2) **a**	2.4 (0.1−10.0)

Means within columns and groups with the same letter are not significantly different (Tukey’s HSD, *P *> 0.05).

^a^Trial 1: *F*_3,18_ = 0.14, *P *= 0.933; Trial 2: *F*_3,16_ = 2.21, *P *= 0.127; Trial 3: *F*_3,20_ = 2.84, *P *= 0.064

^b^Trial 1: *F*_3,20_ = 3.06, *P *= 0.052; Trial 2: *F*_3,20_ = 2.39, *P *= 0.099; Trial 3: *F*_3,20_ = 1.23, *P *= 0.324

^c^Trial 1: *F*_3,20_ = 0.26, *P *= 0.853; Trial 2: *F*_3,20_ = 1.31, *P *= 0.342; Trial 3: *F*_3,20_ = 7.14, *P *= 0.002

^d^Trial 1: *F*_3,20_ = 1.29, *P *= 0.305; Trial 2: *F*_3,20_ = 1.23, *P *= 0.303; Trial 3: *F*_3,20_ = 0.63, *P *= 0.606

**Table 2:** Response of *Drosophila suzukii* flies when given a choice of a cotton wick with raspberry juice or a wick with juice and an essential oil.

Essential oil	Fly sex	*N*	Slope ± SE	EC95 (mg wick^−1^)	95% CL	χ^2^^a^
Peppermint	Male	24	0.34 **±** 0.07	9.9	8.2 – 13.7	0.55
	Female	25	0.31 **±** 0.09	8.7	6.0 – 18.2	1.67
Geranium	Male	25	0.40 **±** 0.11	7.5	5.3 – 14.9	1.68
	Female	33	0.41 **±** 0.07	6.8	5.2 – 10.0	0.77

EC95 is the effective concentration required to achieve 95% repellency.

^a^No significant deviations form the probit model at α = 0.05

**Table 3:** Volatility as measured by total weight lost and release rates for 12 essential oils in 2 mL vials with 3 mm holes in lids held in a fume hood at 22 °C ± 1 °C.

Essential oil	Density (mg μL^−1^)	Decrease in weight (mg)^a^		Release rate (mg h^−1^)		
		96 h		0–6 h	6–24 h	24–96 h
Peppermint	0.84	4.1	**g**	0.07	0.05	0.05
Geranium	0.92	7.8	**fg**	0.23	0.12	0.06
Citronella	0.81	11.9	**efg**	0.78	0.22	0.06
Lavender	0.76	13.7	**defg**	0.89	0.15	0.08
Ginger	0.85	18.5	**def**	0.27	0.21	0.18
Eastern white cedar	0.85	24.0	**de**	0.42	0.31	0.22
Thyme	0.79	24.7	**d**	1.17	0.21	0.19
Eucalyptus	0.91	36.7	**c**	0.60	0.38	0.36
Rosemary	0.82	46.6	**bc**	0.73	0.40	0.43
White spruce	0.89	50.1	**b**	0.66	0.46	0.64
Balsam fir	0.74	55.7	**ab**	0.76	0.59	0.56
White pine	0.73	63.6	**a**	1.13	0.65	0.63

^a^Means followed by the same letter are not significantly different, Tukey’s HSD, *P *> 0.05.
